# Pirfenidone controls the feedback loop of the AT1R/p38 MAPK/renin-angiotensin system axis by regulating liver X receptor-α in myocardial infarction-induced cardiac fibrosis

**DOI:** 10.1038/srep40523

**Published:** 2017-01-16

**Authors:** Chunmei Li, Rui Han, Le Kang, Jianping Wang, Yonglin Gao, Yanshen Li, Jie He, Jingwei Tian

**Affiliations:** 1 School of Pharmacy, Key Laboratory of Molecular Pharmacology and Drug Evaluation (Yantai University), Ministry of Education, Collaborative Innovation Center of Advanced Drug Delivery System and Biotech Drugs in Universities of Shandong, Yantai University, Yantai, 264005, P.R. China; 2 School of Life Sciences, Yantai University, Yantai, 264005, P.R. China; 3 Yantai yuhuangding Hospital, Yantai, 264005, P.R. China

## Abstract

Pirfenidone (PFD), an anti-fibrotic small molecule drug, is used to treat fibrotic diseases, but its effects on myocardial infarction (MI)-induced cardiac fibrosis are unknown. The aim of this study was to determine the effects of PFD on MI-induced cardiac fibrosis and the possible underlying mechanisms in rats. After establishment of the model, animals were administered PFD by gavage for 4 weeks. During the development of MI-induced cardiac fibrosis, we found activation of a positive feedback loop between the angiotensin II type 1 receptor (AT1R)/phospho-p38 mitogen-activated protein kinase (p38 MAPK) pathway and renin-angiotensin system (RAS), which was accompanied by down-regulation of liver X receptor-α (LXR-α) expression. PFD attenuated body weight, heart weight, left ventricular weight, left ventricular systolic pressure, and ±dp/dt_max_ changes induced by MI, which were associated with a reduction in cardiac fibrosis, infarct size, and hydroxyproline concentration. Moreover, PFD inhibited the AT1R/p38 MAPK pathway, corrected the RAS imbalance [decreased angiotensin-converting enzyme (ACE), angiotensin II, and angiotensin II type 1 receptor expression, but increased ACE2 and angiotensin (1-7) activity and Mas expression] and strongly enhanced heart LXR-α expression. These results indicate that the cardioprotective effects of PFD may be due, in large part, to controlling the feedback loop of the AT1R/p38 MAPK/RAS axis by activation of LXR-α.

Cardiac fibrosis contributes to significant morbidity and mortality worldwide. Although various therapeutic strategies have been developed to treat this condition, cardiac fibrosis is clinically variable, and the underlying mechanism is complex and remains intractable. The renin-angiotensin system (RAS) is a major pathway in cardiac fibrosis and myocardial infarction (MI). The RAS consists of two counter-regulatory axes that control cardiovascular functions. The first axis consists of a series of enzymatic reactions culminating in the generation of angiotensin II (Ang II), which can result in angiotensin II type 1 receptor (AT1R)-dependent MI and cardiac fibrosis by activation of the angiotensin-converting enzyme (ACE)-Ang II-AT1R axis^1^,^2^. The second axis is the ACE2-angiotensin(1-7) [Ang(1-7)]-Mas pathway that acts as a physiological antagonist of the ACE-Ang II-AT1R axis. The balance of the ACE/ACE2 ratio and therefore the RAS (Fig. 1) is critical for the pathogenesis of cardiac fibrosis and myocardial hypertrophy^3^.

Mitogen-activated protein kinases (MAPKs) are involved in various processes that contribute to heart failure. p38 MAPK, a major member of the MAPKs, has been shown to play a vital role in the development of cardiac fibrosis, MI, and cardiac hypertrophy^4^. Recent studies have suggested the involvement of the AT1R/p38 MAPK pathway in pancreatic fibrosis^5^, renal tubulointerstitial fibrosis^6^, and peritoneal fibrosis^7^. Importantly, the AT1R/p38 MAPK pathway also affects the RAS by modulation of the ACE/ACE2 ratio^8^. These findings indicate a regulatory mechanism that operates between the AT1R/p38 MAPK pathway and RAS in the development of fibrotic disease.

Liver X receptor-α (LXR-α) is a member of the nuclear receptor family of transcription factors and is an important regulator of cholesterol, fatty acids, and glucose homeostasis. Recently, LXR-α was reported to be a new target for treatment of cardiac remodelling and myocardial hypertrophy^9^,^10^. Interestingly, a growing number of studies have demonstrated that LXR-α not only inhibits the ACE-Ang II-AT1R axis in isoproterenol-induced animal heart failure^11^, but also reduces phospho-p38 MAPK expression in leptin-induced liver fibrosis^12^. In these previous studies, researchers hypothesised that there is crosstalk among the AT1R/p38 MAPK pathway, RAS, and LXR-α. However, it is unclear whether this mechanism is also involved in cardiac fibrosis.

In the current study, we used an MI-induced rat model of cardiac fibrosis. The results showed that myocardial injury activated the AT1R/p38 MAPK pathway that disrupted the ACE/ACE2 ratio and further imbalanced the ACE-Ang II-AT1R and ACE2-Ang(1-7)-Mas axes (including increases in ACE, Ang II, and AT1R, and decreases in ACE2, Ang(1-7), and Mas). Moreover, increasing Ang II and decreasing Ang(1-7) synergistically inhibited LXR-α expression. Consequently, the decrease of LXR-α further activated the AT1R/p38 MAPK pathway. This signalling created a positive feedback loop that amplified AT1R/p38 MAPK signalling, thereby disturbing the RAS balance and inducing cardiac fibrosis (Fig. 2). Interestingly, pirfenidone (5-methyl-1-phenyl-2- [1 H]-pyridone, PFD) activated LXR-α expression, inhibited the AT1R/p38 MAPK pathway, and balanced the RAS in this rat model of cardiac fibrosis (Fig. 2).

PFD is a novel anti-fibrotic agent that has shown promising results in various models and clinical trials^13^,^14^. Cumulative evidence indicates the anti-fibrotic potential of PFD *via* inhibition of ACE and phospho-p38 MAPK in renal fibrosis and lung fibrosis, respectively^15^,^16^. To determine the role and mechanism underlying the anti-fibrotic property of PFD, we established a rat model of cardiac fibrosis to evaluate the AT1R/p38 MAPK pathway, RAS, and LXR-α expression. Our results revealed that PFD protected against cardiac fibrosis, which may be partially controlled by the feedback loop of the AT1R/p38 MAPK/RAS axis *via* LXR-α activation.

## Results

### Effects of PFD on MI-induced cardiac hypertrophy and left ventricular systolic dysfunction

To assess the effects of PFD on heart failure, we administered PFD to MI rats for 4 weeks and evaluated cardiac hypertrophy and functions. As shown in Table 1, the heart weight (HW), left ventricle weight (LVW), HW to body weight ratio (HW/BW, mg/g), and LVW to body weight ratio (LVW/BW, mg/g) were significantly increased in MI rats after 4 weeks compared with the sham group. Additionally, the left ventricular end-diastolic pressure (LVEDP) was increased, while the left ventricular systolic pressure (LVSP) and maximum rate of increase/decrease of left ventricle pressure (±dP/dt_max_) were decreased in MI rats (Table 2). These results indicated that cardiac hypertrophy and dysfunction were already present 4 weeks after MI. We administered 20 mg/kg losartan and 300 mg/kg PFD to rats, and the results indicated that losartan and PFD restored LVSP and ±dP/dt _max_ to near normal levels (P < 0.01 and P < 0.05). Moreover, the HW and LVW were decreased compared with the model group (P < 0.01 and P < 0.05), suggesting that the drugs exerted cardioprotective effects by regulation of systolic and diastolic cardiac functions during the chronic phase of MI-induced heart failure. In addition, PFD and losartan decreased HW/BW and LVW/BW ratios, although these differences were not statistically significant (Table 1).

### Effects of PFD on MI-induced cardiac fibrosis and infarct size

**Figure 3:**
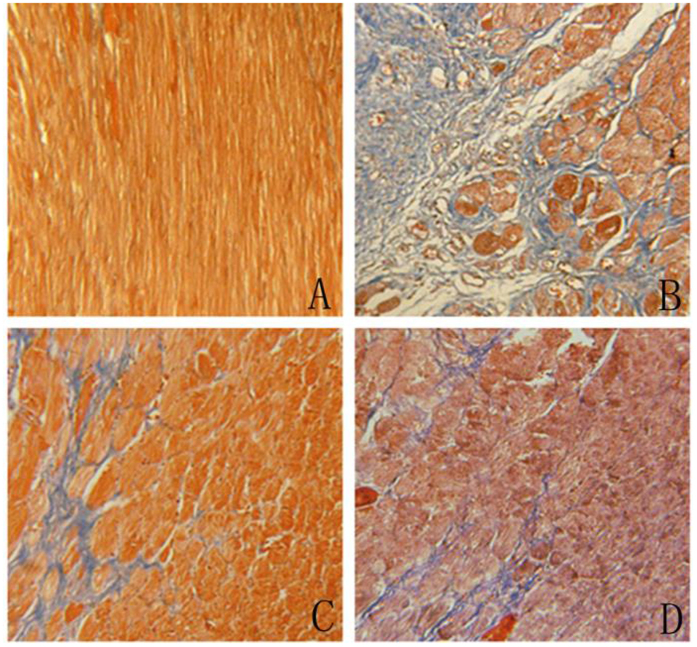
Effects of PFD on MI-induced cardiac fibrosis (×200). (**A**) Sham group, (**B**) model group, (**C**) PFD group, and (**D**) losartan group. Data are reported as means ± SEM (n = 13 for sham group, 12 for model group, 13 for PFD group, and 13 for losartan group). Differences between groups were examined by ANOVA followed by Dunnett’s test. ^#^P < 0.05, ^##^P < 0.01 *vs.* model group.

**Figure 4:**
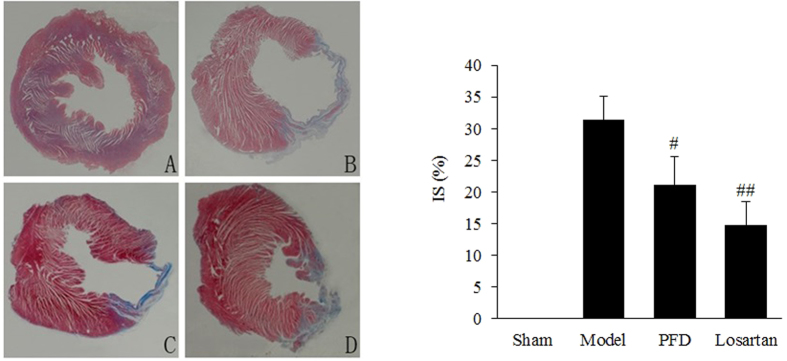
Effects of PFD on MI-induced infarct size (IS) (×10). (**A**) Sham group, (**B**) model group, (**C**) PFD group, and (**D**) losartan group. Data are reported as means ± SEM (n = 13 for sham group, 12 for model group, 13 for PFD group, and 13 for losartan group). Differences between groups were examined by ANOVA followed by Dunnett’s test. **P < 0.01 *vs.* Sham group. ^#^P < 0.05, ^##^P < 0.01 *vs.* model group.

**Figure 5:**
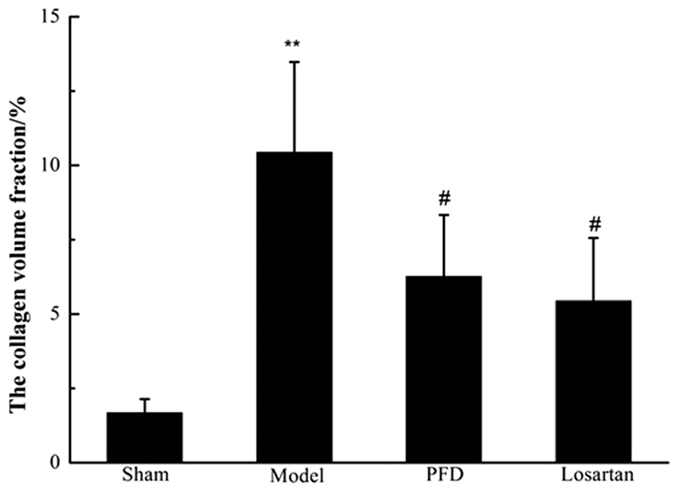
Effects of PFD on MI-induced CVF (n = 13 for sham group, 12 for model group, 13 for PFD group, and 13 for losartan group). Data are reported as means ± SEM. Differences between groups were examined by ANOVA followed by Dunnett’s test. **P < 0.01 *vs.* sham group. ^#^P < 0.05 *vs.* model group.

We used Masson’s trichrome staining to assess cardiac fibrosis and the infarct size. Cardiac fibrosis, especially interstitial fibrosis (collagen staining in blue), was significantly increased in MI hearts compared with sham animal hearts (Fig. 3B). PFD treatment substantially reduced these lesions (Fig. 3C), and losartan also ameliorated these pathological changes (Fig. 3D). Image and quantitative analyses indicated that the cardioprotective effects against cardiac fibrosis in losartan- and PFD-treated rats were consistent with a smaller infarct size (P < 0.05 and P < 0.01; Fig. 4). Moreover, the collagen volume fraction (CVF) in MI model, losartan, and PFD groups were 10.44 ± 3.04%, 5.44 ± 2.12% (P < 0.05), and 6.26 ± 2.07% (P < 0.05), respectively (Fig. 5). These results also strongly supported the cardioprotective effects of PFD on MI-induced cardiac fibrosis.

### Effects of PFD on fibrosis-related proteins in MI rat hearts

**Figure 6:**
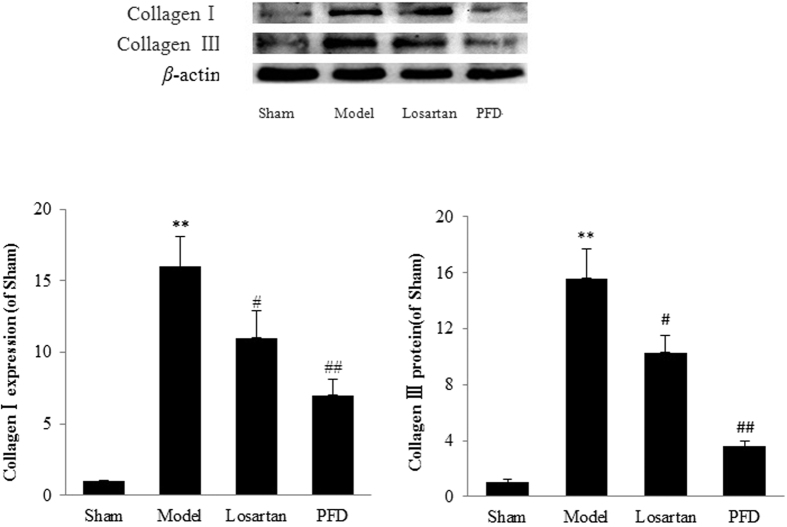
Effects of PFD on collagen I and III expression. Data are reported as means ± SEM (n = 5). Differences between groups were examined by ANOVA followed by Dunnett’s test. *P < 0.05, **P < 0.01 *vs.* sham group. ^#^P < 0.05, ^##^P < 0.01 *vs.* model group. Cropped blots are shown. Full length gels are included in the Supplementary information.

**Figure 7:**
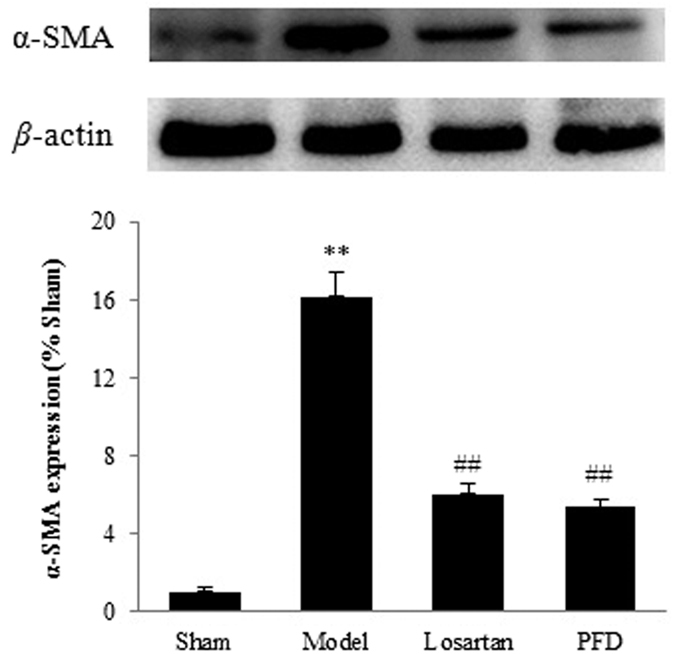
Effects of PFD on α-SMA expression. Data are reported as means ± SEM (n = 5). Differences between groups were examined by ANOVA followed by Dunnett’s test. **P < 0.01 *vs.* sham group. ^#^P < 0.05, ^##^P < 0.01 *vs.* model group. Cropped blots are shown. Full length gels are included in the Supplementary information.

**Figure 8:**
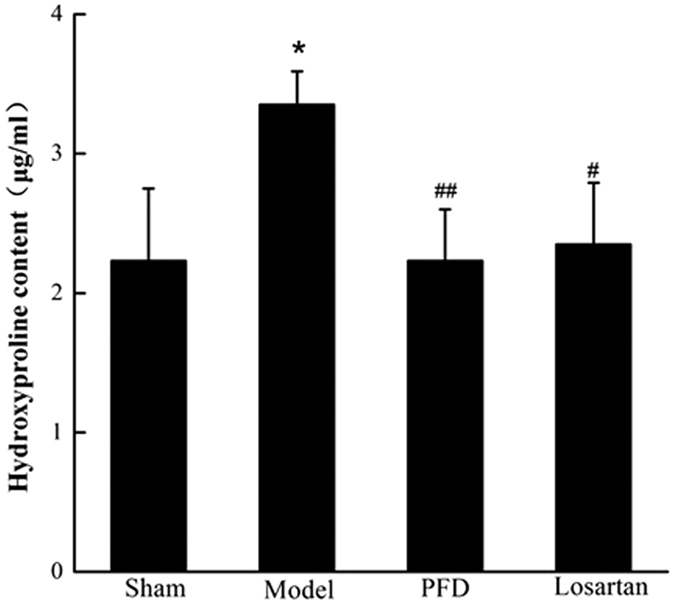
Effects of PFD on hydroxyproline concentrations (n = 13 for sham group, 12 for model group, 13 for PFD group, and 13 for losartan group). Data are reported as means ± SEM. Differences between groups were examined by ANOVA followed by Dunnett’s test. *P < 0.05 *vs.* sham group. ^#^P < 0.05, ^##^P < 0.01 *vs.* model group.

The *in vivo* results showed the ameliorating effects of PFD on cardiac dysfunction and fibrotic progression. To detect the expression levels of fibrosis-related proteins, we performed western blot analyses. The results revealed that fibrosis-associated proteins, such as collagen I, collagen III, and α-smooth muscle actin (α-SMA), were strongly induced in rat hearts of the MI group, whereas they were significantly suppressed by losartan and PFD administration (P < 0.05 and P < 0.01; Figs 6 and 7). Hydroxyproline, a sensitive biochemical marker indicating collagen fibre changes, was also significantly increased after MI, but it was inhibited by losartan and PFD treatment (P < 0.05 and P < 0.01; Fig. 8).

### Effects of PFD on the AT1R/p38 MAPK pathway

**Figure 9:**
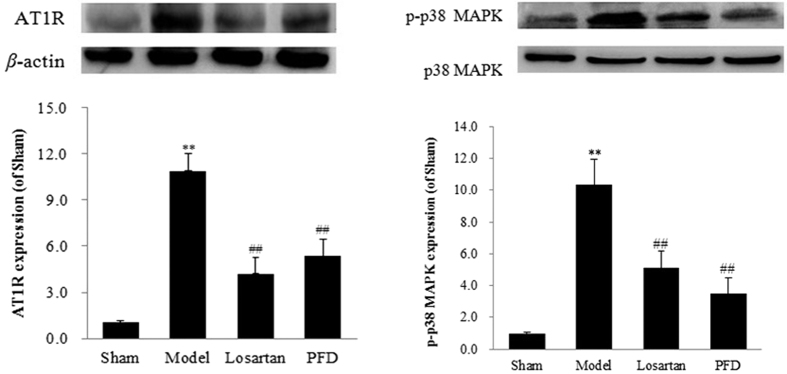
Effects of PFD on AT1R and phospho-p38 MAPK (p-p38 MAPK) expression. Data are reported as means ± SEM (n = 5). Differences between groups were examined by ANOVA followed by Dunnett’s test. *P < 0.05, **P < 0.01 *vs.* sham group. ^#^P < 0.05, ^##^P < 0.01 *vs.* model group. Cropped blots are shown. Full length gels are included in the Supplementary information.

Because of the role of the AT1R/p38 MAPK pathway in cardiac fibrosis, we assessed the levels of AT1R and phospho-p38 MAPK. As shown in Fig. 9, compared with the control group, the expression of AT1R and phospho-p38 MAPK was increased by 10.90 ± 1.12% and 10.33 ± 1.61% in the model group, respectively (P < 0.01). However, the AT1R blocker losartan notably inhibited this pathway, as shown by the decrease in AT1R and phospho-p38 MAPK expression (AT1R, 4.20 ± 1.05%; phospho-p38 MAPK, 5.12 ± 1.05%; all P < 0.01). PFD also normalised the expression of these two proteins compared with the model group (AT1R, 5.35 ± 1.07%; phospho-p38 MAPK, 3.49 ± 1.00%; all P < 0.01).

### Effects of PFD on ACE-Ang II-AT1R and ACE2-Ang(1-7)-Mas axes

**Figure 10:**
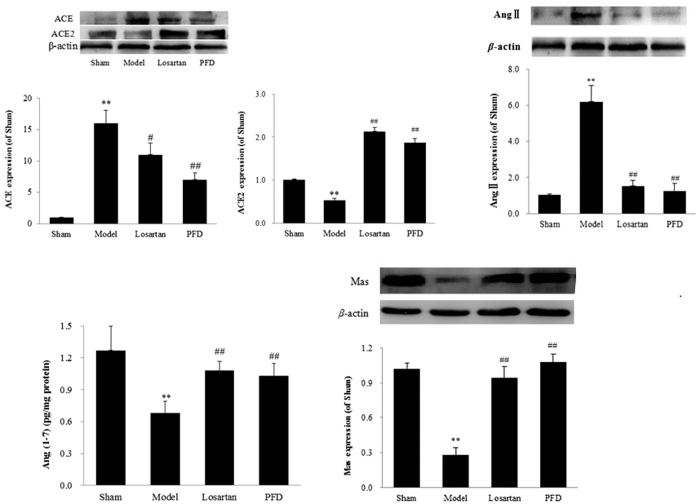
Effects of PFD on ACE, ACE2, Ang II, Ang(1-7), and Mas expression. Data are reported as means ± SEM (n = 5). Differences between groups were examined by ANOVA followed by Dunnett’s test. *P < 0.05, **P < 0.01 *vs.* sham group. ^#^P < 0.05, ^##^P < 0.01 *vs.* model group. Cropped blots are shown. Full length gels are included in the Supplementary information.

The balance between ACE-Ang II-AT1R and ACE2-Ang(1-7)-Mas axes is critical in the pathogenesis of cardiac fibrosis and myocardial hypertrophy. In the present study, we measured the related proteins. As shown in Figs 9A and 10, compared with the sham group, Ang II, ACE, and AT1R expression was markedly increased, and the expression of ACE2, Ang(1-7), and Mas was down-regulated (P < 0.05 and P < 0.01). As expected, all of these changes were ameliorated by PFD administration. Additionally, losartan, an AT1R blocker, not only inhibited the ACE-Ang II-AT1R axis but also activated the ACE2-Ang(1-7)-Mas axis. These results showed that PFD treatment strongly influences RAS-related protein expression in MI-induced cardiac failure.

### Effects of PFD on LXR-α expression

**Figure 11:**
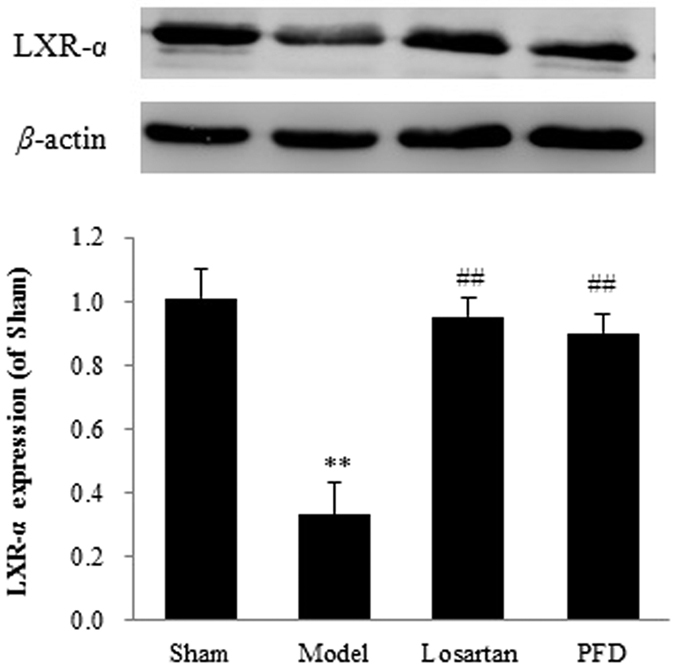
Effects of PFD on LXR-α expression (n = 5). Data are reported as means ± SEM. Differences between groups were examined by ANOVA followed by Dunnett’s test. **P < 0.01 *vs.* sham group. ^##^P < 0.01 *vs.* model group. Cropped blots are shown. Full length gels are included in the Supplementary information.

Western blotting of proteins extracted from the left ventricular of rats revealed a substantial decrease in LXR-α after MI. However, LXR-α expression was significantly up-regulated compared with the MI model group after 4 weeks of PFD administration (P < 0.01, Fig. 11). Therefore, the protective effects of PFD could be due, in large part, to activation of LXR-α. Losartan also activated LXR-α expression in animal hearts during MI-induced cardiac fibrosis.

## Discussion

Cardiac fibrosis is a critical pathological change in the development of heart failure caused by MI^17^. Although several anti-fibrotic drugs (such as β-blockers, calcium channel blockers, ACE inhibitors, and angiotensin receptor blockers) have been used in the clinic, the unsatisfactory efficacy and long-term safety concerns of current therapies necessitate the identification of new targets to effectively prevent and treat cardiac fibrosis. There is an urgent need for novel treatments of this disease. PFD, a small molecule drug, has universal anti-fibrotic effects in various types of fibrotic diseases^14^,^18^.

Yamazaki *et al*. found that PFD treatment results in a significant reduction of left ventricular hypertrophy and cardiac fibrosis in an Ang II-induced mouse hypertrophic model^14^, suggesting that the RAS may be a novel target of PFD for treatment of cardiomyopathy. However, the underlying molecular mechanisms remained unknown. In this study, for the first time, we showed that PFD balances the RAS to prevent MI-induced cardiac fibrosis.

The RAS includes two counter-regulatory axes, ACE-Ang II-AT1R and ACE2-Ang(1-7)-Mas, which are important for the formation and development of cardiac fibrosis in MI and chronic heart failure^19^. Accumulating evidence suggests that increases in ACE are detrimental to the heart, because they result in impaired contractility and cardiac hypertrophy due in part to the inhibition of ACE2-mediated cardioprotection^20^,^21^. ACE inhibitors and AT1R blockers also increase myocardial ACE2 levels and activity in the clinic^22^. Additionally, ACE2 overexpression protects against ACE-mediated cardiac hypertrophy and cardiac fibrosis^23^. Thus, ACE and ACE2 may regulate each other by feedback inhibition. Our study supports this hypothesis, and the results suggested that the ACE/ACE2 ratio was disrupted during the development of cardiac fibrosis. In addition, the ACE-Ang II-AT1R axis was notably activated as shown by high levels of ACE, Ang II, and AT1R in the heart tissue. However, the ACE2-Ang(1-7)-Mas axis was inhibited, which acts as a physiological antagonist. PFD and losartan rescued the ACE/ACE2 ratio and balanced the RAS. Losartan is an Ang II receptor antagonist with an antihypertensive activity predominantly due to selective inhibition of AT1R and consequentially reduced pressor effect of Ang II. It is used in the treatment of hypertension and heart failure. It has also been used to reduce the risk of stroke in patients with left ventricular hypertrophy and in the management of MI^24^. Recently, Wang *et al*. observed that losartan effectively inhibits pressure overload-induced cardiac remodelling by up-regulating ACE2 expression and down-regulating ACE expression^25^. Taken together, the previous studies and our results indicate similar cardioprotection of PFD and losartan, which may be attributed to the effects on RAS axes by alteration of the ACE/ACE2 ratio.

In terms of how PFD modulates the ACE/ACE2 ratio, we hypothesised that the AT1R/p38 MAPK signalling pathway might play an important role. AT1R/p38 MAPK, an important signalling pathway, is involved in pancreatic fibrosis, renal tubulointerstitial fibrosis, and peritoneal fibrosis. Moreover, previous studies have reported that activation of the AT1R/p38 MAPK pathway induces an imbalance in the ACE/ACE2 ratio in HK-2 cells and neurons^7^,^8^.

In the current study, we first found up-regulation of AT1R and p38 MAPK in fibrotic hearts, which was associated with an increase in ACE and a decrease in ACE2. Furthermore, losartan strongly ameliorated the expression of these proteins. Based on previous studies^7^,^8^ and our results, we confirmed that activation of the AT1R/p38 MAPK pathway altered the RAS by up-regulation of the ACE/ACE2 ratio, which increased ACE, Ang II, and AT1R, but decreased ACE2, Ang(1-7) and Mas. Furthermore, AT1R overexpression amplified AT1R/p38 MAPK signalling, thus creating a positive feedback loop. Interestingly, PFD also inhibited AT1R and p38 MAPK expression, and corrected the ACE/ACE2 ratio. Taking these findings together, we conclude that PFD controls the AT1R/p38 MAPK signalling pathway, corrects the ACE/ACE2 ratio, balances the RAS, and then ameliorates MI-induced cardiac fibrosis.

LXR-α has been identified as a novel therapeutic target for fibrotic diseases such as dermal fibrosis and liver fibrosis^26^,^27^. A recent study showed that treatment of rats with synthetic LXR-α interferes with the Ang II-mediated pressor response^28^. Cannon *et al*. also reported that an LXR-α agonist significantly improves transverse aortic constriction-induced cardiac dysfunction and cardiac fibrosis *in vivo*^29^. Furthermore, Ang II and Ang(1-7), two important elements of the RAS, inhibit and activate LXR-α expression, respectively^30^,^31^. Kuipers *et al*. found that T09, an LXR-α agonist, decreases AT1R and p38 MAPK in wild-type mice but not in LXR-α^−/−^ mice^11^,^32^. Therefore, the authors speculated that LXR-α plays a vital role in the positive feedback loop between the AT1R/p38 MAPK pathway and RAS. In the present study, we found a significant decrease in the LXR-α expression of animal hearts after MI-induced cardiac fibrosis, which was associated with activation of the AT1R/p38 MAPK pathway and dysfunction of the RAS. However, losartan inhibited the AT1R/p38 MAPK pathway and activated LXR-α expression. These results were consistent with a previous study in which losartan up-regulated LXR-α mRNA expression in human monocyte-macrophages^33^. These results indicate that the positive feedback loop between the AT1R/p38 MAPK pathway and RAS is influenced by inhibition of LXR-α activity. Furthermore, PFD not only blocked the AT1R/p38 MAPK pathway and corrected the RAS balance, but also substantially increased LXR-α activity in MI-induced cardiac fibrosis. These results indicate that the cardioprotection of PFD could be due, in large part, to controlling the feedback loop of the AT1R/p38 MAPK/RAS axis by activation of LXR-α.

One limitation of our study is that, although we demonstrate that PFD treatment activates LXR-α and controls the feedback loop of the AT1R/p38-MAPK/RAS axis in a rat model of MI-induced cardiac fibrosis *in vivo*, further studies should address this issue with gain- or loss-of-function assays *in vitro*. Moreover, this aspect may be addressed using genetically altered mice with deficiencies in LXR-α or through the use of small interfering RNA, which is currently underway.

In summary, our present study provides *in vivo* confirmation that the positive feedback loop between the AT1R/p38 MAPK pathway and RAS is influenced by inhibition of LXR-α activity, offering a new understanding of human fibrotic diseases. Additionally, we demonstrated for the first time that one of the major mechanisms of PFD may be mediated by the feedback loop of the AT1R/p38 MAPK/RAS axis, partially *via* activation of LXR-α expression. This study suggests that LXR-α may be a new target of PFD for fibrotic disease therapy.

## Materials and Methods

This study was approved by the Ethics Committee of Yantai University. All animal protocols were in accordance with the guidelines on humane use and care of laboratory animals for biomedical research published by the NIH (No. 85–23, revised 1996).

### Chemicals and reagents

PFD was purchased from Wuhan Kang Bao Tai Biotech Co. Ltd. (Wuhan, China). Losartan was purchased from Xi’an Kaihong Biological Technology Co., Ltd. (Xi’an, China). A Masson trichrome staining kit was purchased from Maixin Biotech. Co., Ltd. (Shanghai, China). Anti-LXR-α, -phospho-p38 MAPK, -p38 MAPK, -Mas, -AT1R, -ACE2, and -ACE antibodies were obtained from Santa Cruz Biotechnology (CA, USA). Anti-β-actin, -collagen I, -collagen III, and -Ang II antibodies were obtained from Abcam (Cambridge, UK).

### Animals and surgical preparation

Male Sprague-Dawley rats (260 ± 20 g) were provided by the Experimental Animal Center of Shandong Luye Pharmaceutical Co., Ltd. (specific pathogen-free grade). All rats were housed in cages under hygienic conditions with a 12-h light/dark cycle at 23 ± 3 °C and 40–60% humidity for 6 days before experiments. The animals were provided with a commercial standard rat cube diet and water *ad libitum*.

An MI model was established by ligation of the left coronary artery as described in our previous study^34^. In brief, animals were anaesthetised by injection of sodium pentobarbital (35 mg/kg, i.p.) and artificially ventilated using a volume-regulated respirator. The heart was exposed, and the left coronary artery was ligated at 2–3 mm from its origin between the left atrium and pulmonary artery conus using a 6-0 prolene suture. A successful operation was confirmed by the occurrence of ST-segment elevation in an electrocardiogram. This operation was performed by an experimenter who was blinded to the group assignments of the animals. The sham-operated group underwent thoracotomy and cardiac exposure without coronary ligation (n = 13). After establishment of the model, animals were divided into model (n = 13), PFD (300 mg/kg; n = 13), and losartan (20 mg/kg; n = 13) groups. Test substances were administrated by gavage daily for 4 weeks.

### Cardiac function assessment

Animals were anaesthetised by injection of sodium pentobarbital (35 mg/kg, i.p.), and their cardiac functions were assessed by invasive haemodynamic evaluation methods as described in a previous study^34^. Under anaesthesia, the right carotid artery was isolated, and a micromanometer-tipped catheter (Model SPR-838; Millar Instruments) was inserted into the left ventricular cavity through the carotid artery. After a 10-min equilibrium period, haemodynamic parameters, including LVSP, +dP/dt_max_, −dP/dt_max_, and LVEDP, were measured. Moreover, HW and LVW weight were determined to calculate the organ index.

### Masson’s trichrome staining for assessment of cardiac fibrosis and infarct size

To assess the cardioprotective role of PFD on cardiac fibrosis, the collagenous fibrotic area of the heart was determined by Masson’s trichrome staining of 4 μm-thick paraffin-embedded sections. Briefly, the sections were deparaffinised in Histo-Clear and rehydrated by sequential passage through 70–100% ethanol solutions for 5–6 min each followed by washing in distilled water three times. The sections were stained with Masson’s trichrome for 4–5 min. The sections were washed and stained with phosphomolybdic acid for 4–5 min, an aniline blue solution for 4–5 min, and then differentiated for 60 s. After a final wash, the sections were dehydrated using 95% and 100% alcohol solutions followed by dehydration and mounting. The sections were digitally imaged on an Olympus DP25, and the CVF in the peri-infarcted areas was evaluated as a percentage of the fibrotic area (blue staining) to the left ventricular area in an average of five sections of each heart (NIH Image software).

Infarct size was determined using a previously described method^35^. In this method, sections were stained with Masson’s trichrome. Total infarct circumference was calculated as the sum of the endocardial and epicardial infarct lengths from all sections, which were traced manually in the digital images and measured automatically by a computer. Total left ventricular circumference was calculated as the sum of the endocardial and epicardial segment lengths from all sections. Infarct size was calculated as the total infarct circumference divided by the total left ventricular circumference.

### Western blot analysis

Proteins were separated by sodium dodecyl sulfate-polyacrylamide gel electrophoresis, transferred to a nitrocellulose membrane, and incubated with antibodies against β-actin, α-SMA, collagen I, collagen III, Ang II, ACE, ACE2, AT1R, Mas, LXR-α, p38 MAPK, or phospho-p38 MAPK at 4 °C overnight. Labelled proteins were detected with horseradish peroxidase-conjugated secondary antibodies and visualised using the enhanced chemiluminescence method.

### Enzyme-linked immunosorbent assay

Hearts were homogenised for Ang(1-7) analysis by enzyme-linked immunosorbent assays using commercially available kits, according to the manufacturer’s instructions (Jiancheng, Nanjing, China).

### Statistical analyses

Data are reported as the mean ± standard error of the mean (SEM). Statistical analyses were performed using SPSS 17.0 software. Differences between groups were determined by analysis of variance (ANOVA) followed by Dunnett’s test. P < 0.05 was considered as statistically significant.

## Additional Information

**How to cite this article**: Li, C. *et al*. Pirfenidone controls the feedback loop of the AT1R/p38 MAPK/renin-angiotensin system axis by regulating liver X receptor-α in myocardial infarction-induced cardiac fibrosis. *Sci. Rep.* **7**, 40523; doi: 10.1038/srep40523 (2017).

**Publisher's note:** Springer Nature remains neutral with regard to jurisdictional claims in published maps and institutional affiliations.

## Supplementary Material

Supplementary Information

## Figures and Tables

**Figure 1:**
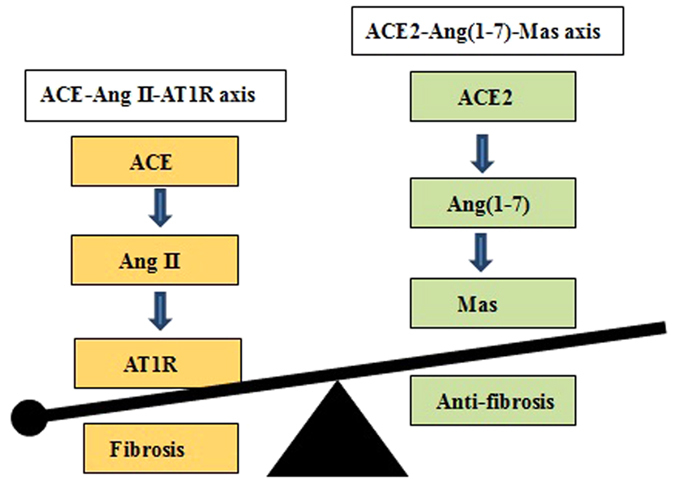
Balance between ACE-Ang II-AT1R and ACE2-Ang(1-7)-Mas axes in the development of cardiac fibrosis.

**Figure 2:**
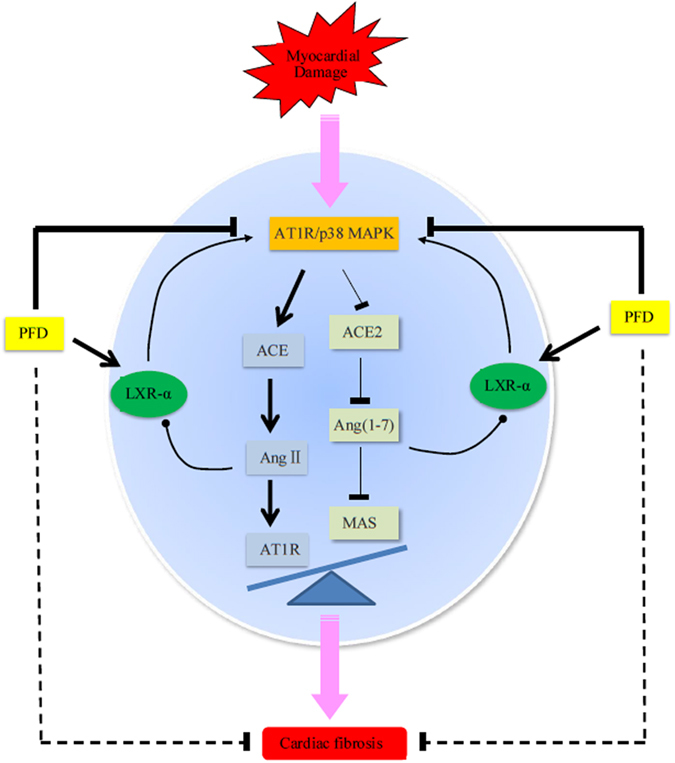
LXR-α involved in the feedback loop of AT1R/p38 MAPK-RAS axis and the interventional effect of PFD. Myocardial injury activated the AT1R/p38 MAPK pathway that disrupted the ACE/ACE2 ratio and further imbalanced ACE-Ang II-AT1R and ACE2-Ang(1-7)-Mas axes, including increases in ACE, Ang II, and AT1R and decreases in ACE2, Ang(1-7) and Mas. Moreover, increasing Ang II and decreasing Ang(1-7) synergistically inhibited LXR-α expression. Consequently, the decrease in LXR-α further activated the AT1R/p38 MAPK pathway. This signalling created a positive feedback loop that amplified AT1R/p38 MAPK signalling, thereby disrupting the RAS balance and inducing cardiac fibrosis. Interestingly, PFD activated LXR-α expression, inhibited the AT1R/p38 MAPK pathway, and balanced the RAS in this rat model of cardiac fibrosis.

**Table 1:** Effects of PFD on cardiac hypertrophy in rats with cardiac fibrosis.

Group	Dose (mg/kg)	BW (g)	HW (mg)	LVW (mg)	HW/BW (mg/g)	LVW/BW (mg/g)
Sham	—	467 ± 32.6	1231 ± 56.3	849.9 ± 40.3	2.6 ± 0.1	1.8 ± 0.1
Model	—	454.8 ± 23.8	1351 ± 77.6^**^	916.5 ± 47.4^**^	2.9 ± 0.1^**^	2.0 ± 0.1^**^
PFD	300	423.7 ± 20.3^##^	1228 ± 66.5^##^	844.8 ± 50.6^##^	2.7 ± 0.1	1.9 ± 0.1
Losartan	20	430 ± 28.8^#^	1237 ± 100.7^##^	853.9 ± 53.1^##^	2.8 ± 0.1	1.9 ± 0.1

Data are reported as means ± SEM (n = 13 for sham group, 12 for model group, 13 for PFD group, and 13 for losartan group). Differences between groups were examined by ANOVA followed by Dunnett’s test. PFD, Pirfenidone; BW, the body weight; HW, heart weight; LVH, left ventricular weight. **P < 0.01 *vs.* sham group. ^#^P < 0.05, ^##^P < 0.01 *vs.* model group.

**Table 2:** Effects of PFD on hemodynamic parameters in rats with cardiac fibrosis.

Group	Dose (mg/kg)	LVSP (mmHg)	LVEDP (mmHg)	+dp/dt_max_ (mmHg/sec)	−dp/dt_max_ (mmHg/sec)
Sham	—	148.1 ± 7.9	5.8 ± 1.1	10806 ± 702	9141 ± 1173
Model	—	115.7 ± 13.4^**^	10.1 ± 2.2^*^	6526 ± 1465^**^	4666 ± 1591^**^
PFD	300	129.3 ± 15.4^#^	11.5 ± 4.2	8663 ± 1596^##^	6475 ± 1414^##^
Losartan	20	134.1 ± 7.7^##^	13.2 ± 3.4	8038 ± 1137^#^	6147 ± 1088^#^

Data are reported as means ± SEM (n = 13 for sham group, 12 for model group, 12 for PFD group, and 12 for losartan group). Differences between groups were examined by ANOVA followed by Dunnett’s test. PFD, Pirfenidone; LVSP, left ventricular systolic pressure; LVEDP, left ventricular end-diastolic pressure. *P < 0.05, **P < 0.01 *vs.* sham group. ^#^P < 0.05, ^##^P < 0.01 *vs.* model group.
