# Supplementary material for: Pirfenidone controls the feedback loop of the AT1R/p38 MAPK/renin-angiotensin system axis by regulating liver X receptor-α in myocardial infarction-induced cardiac fibrosis
Source: Sci Rep. 2017 Jan 16;7:40523. doi: 10.1038/srep40523 (PMC5238375; doi:10.1038/srep40523)

Supplementary information

The manuscript title: Pirfenidone controls the feedback loop of the AT1R/p38 MAPK/renin-angiotensin system axis by regulating liver X receptor-α in myocardial infarction-induced cardiac fibrosis

Author list: Chunmei Li, Rui Han, Le Kang, Jianping Wang, Yonglin Gao,Yanshen Li, Jie He, Jingwei Tian

Supplementary legends: C, Control; M, Model; L, Losartan; B, Pirfenidone

1. **The full-length gels:** Fig.6-Collagen I

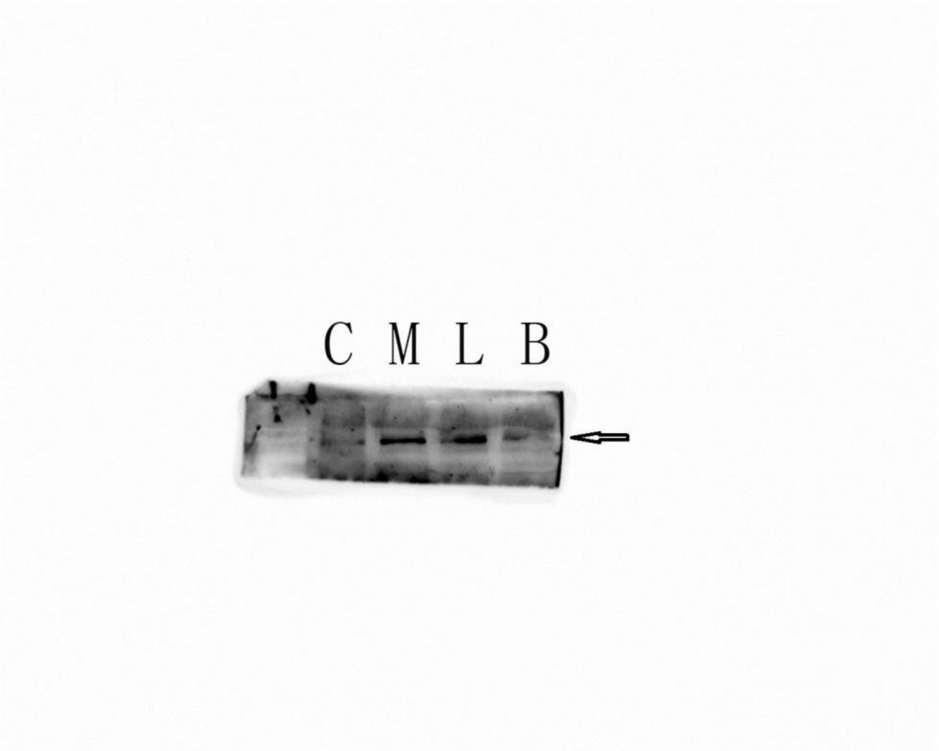

2. **The full-length gels:** Fig.6-Collagen Ш

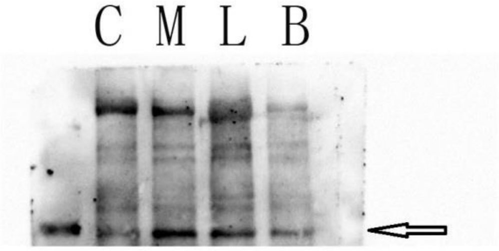

3. **The full-length gels:** Fig.6-Collagen 1 and 3 (β-actin)

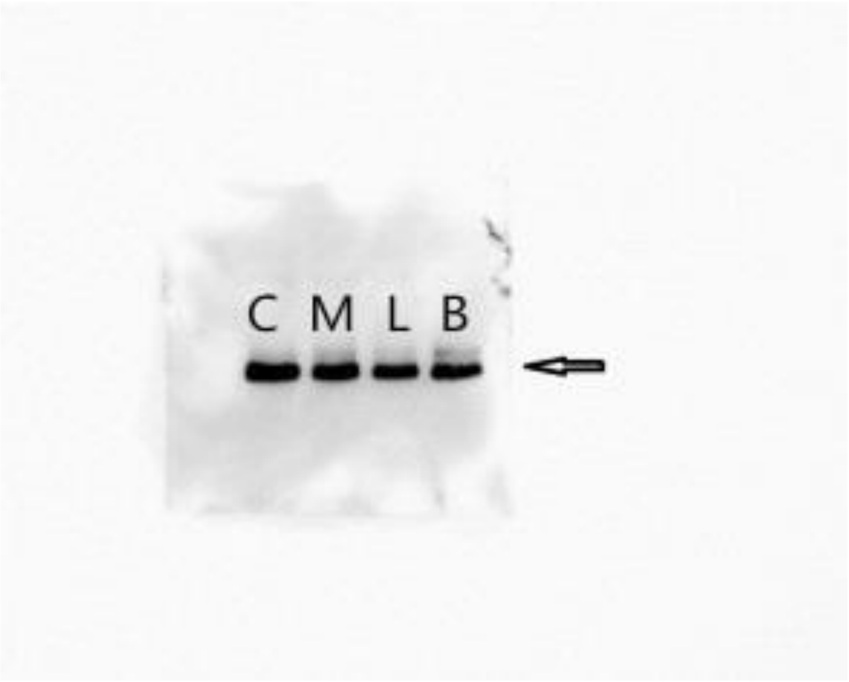

4. **The full-length gels:** Fig.7-α-SMA

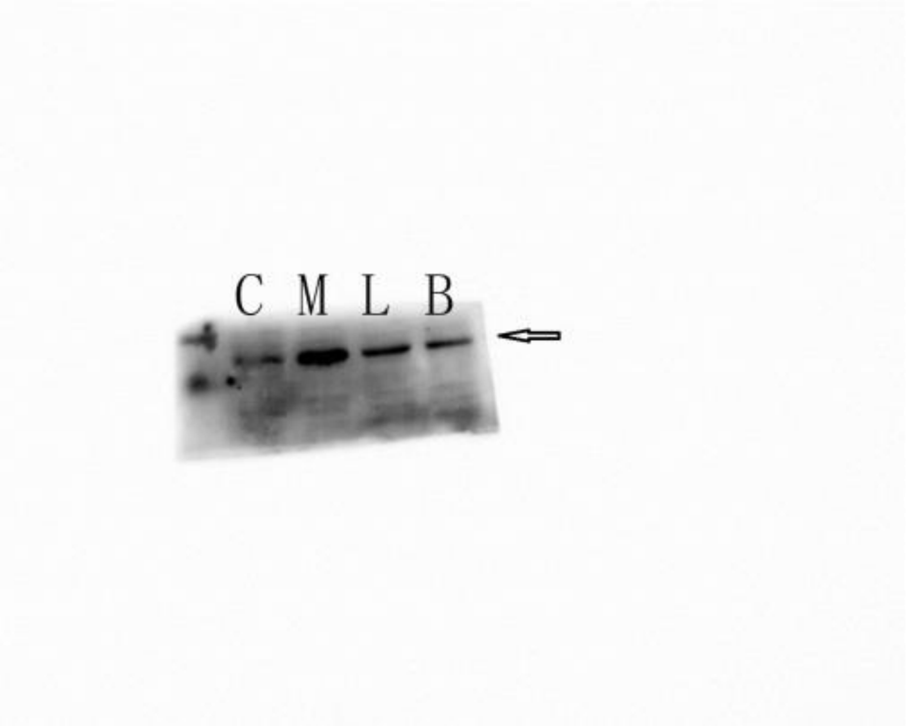

5. **The full-length gels:** Fig.7-α-SMA (β-actin)

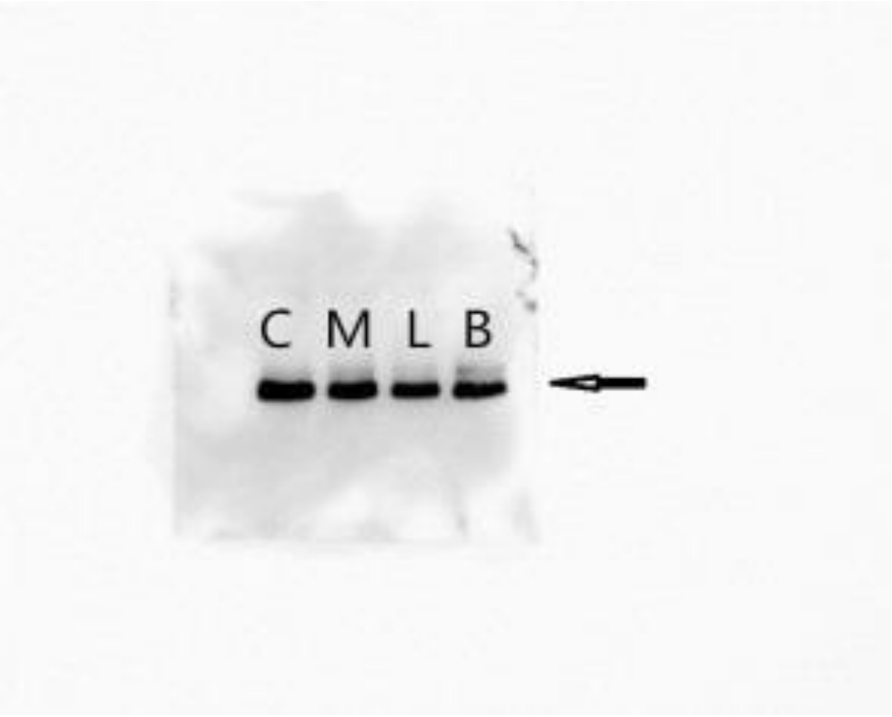

6. **The full-length gels:** Fig.9-AT1R

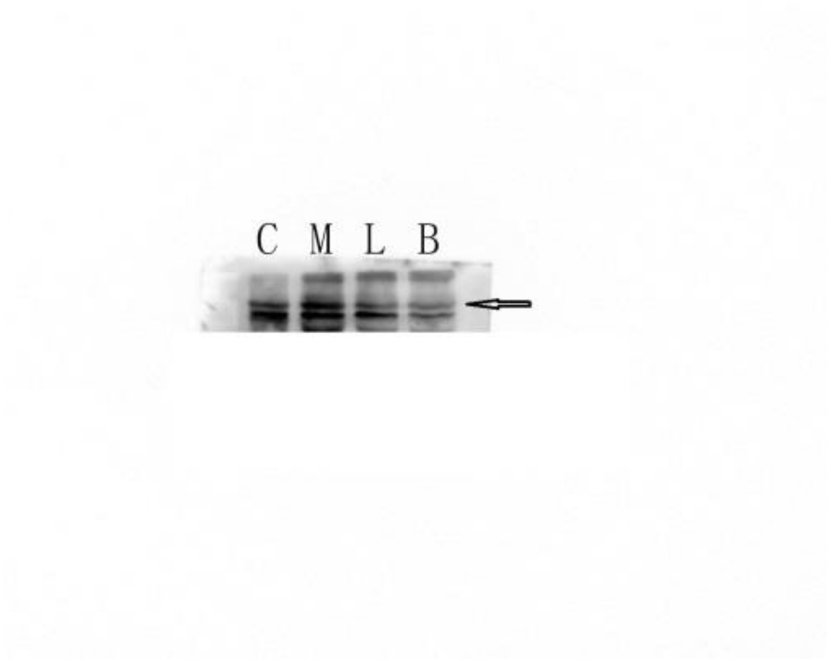

7. **The full-length gels:** Fig.9-AT1R(β-actin)

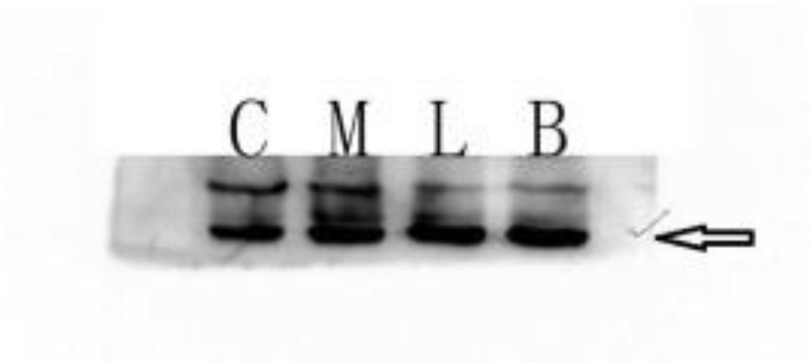

8. **The full-length gels:** Fig.9-P-p38

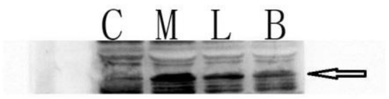

9. The full-length gels: Fig.9-T-p38

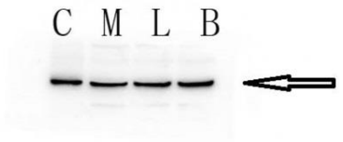

10. The full-length gels: Fig.10-ACE

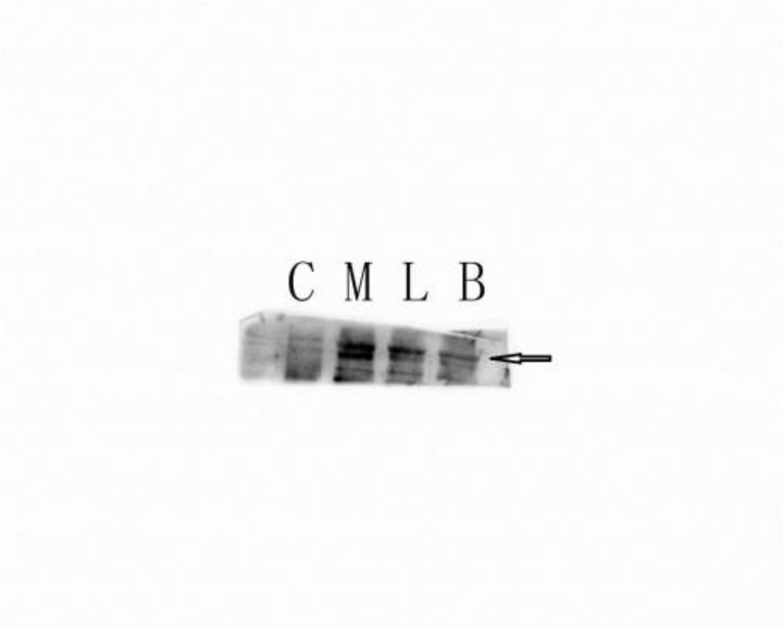

11. **The full-length gels:** Fig.10-ACE2

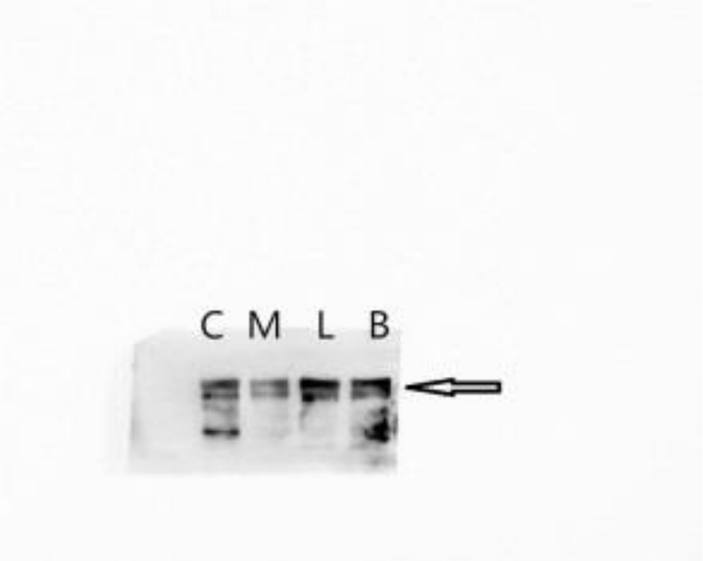

12. **The full-length gels:** Fig.10-ACE ACE2(β-actin）

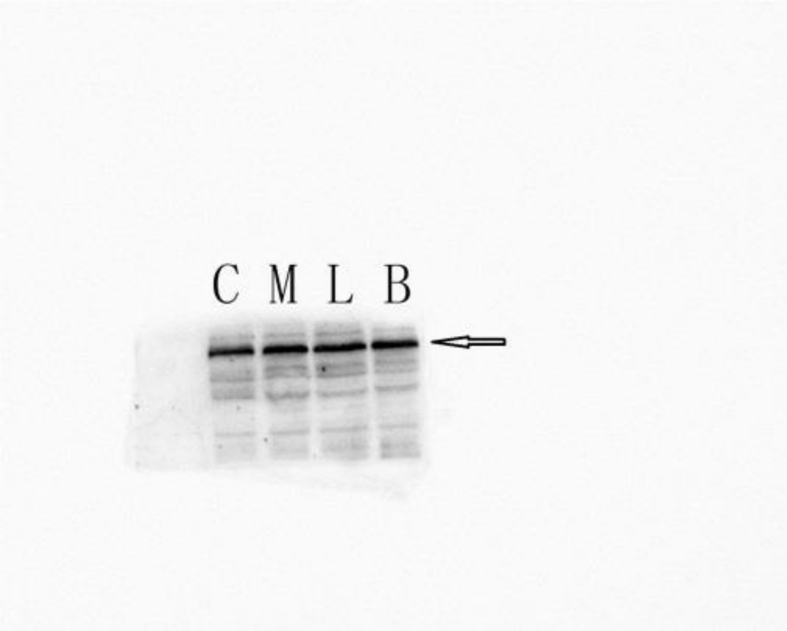

13. The full-length gels: Fig.10-Ang 2

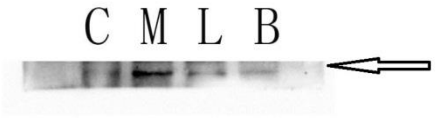

14. The full-length gels: Fig.10-Ang 2(β-actin）

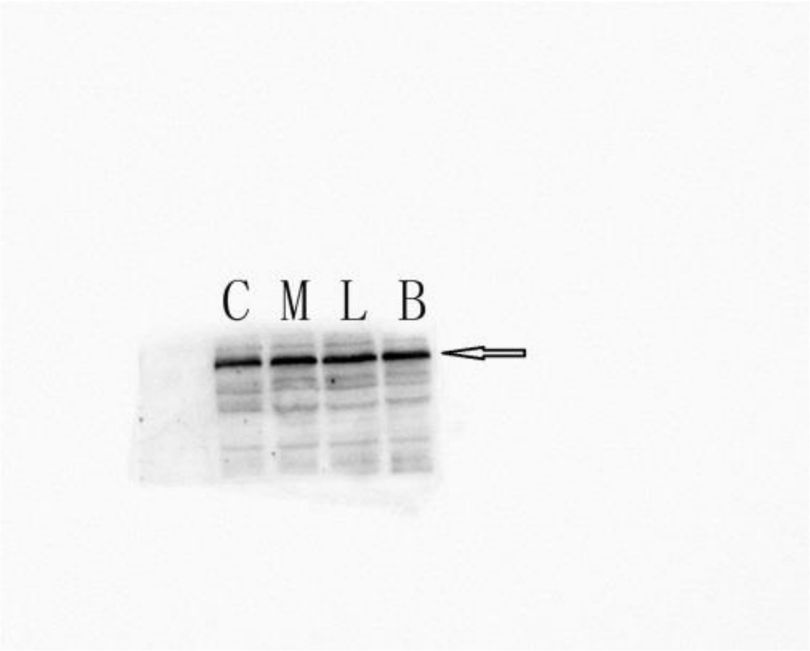

15. The full-length gels: Fig.10-MAS

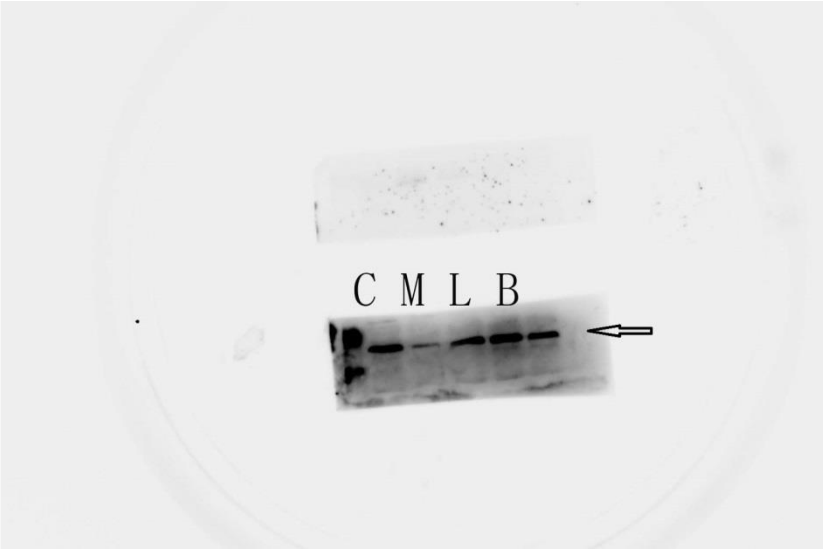

16. The full-length gels: Fig.10-MAS（β-actin）

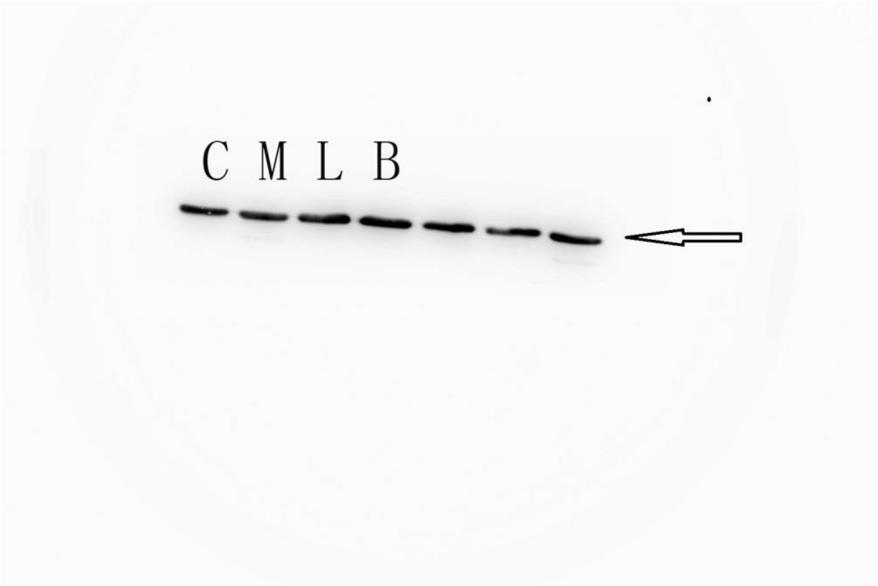

17. The full-length gels: Fig.11-LXR-α

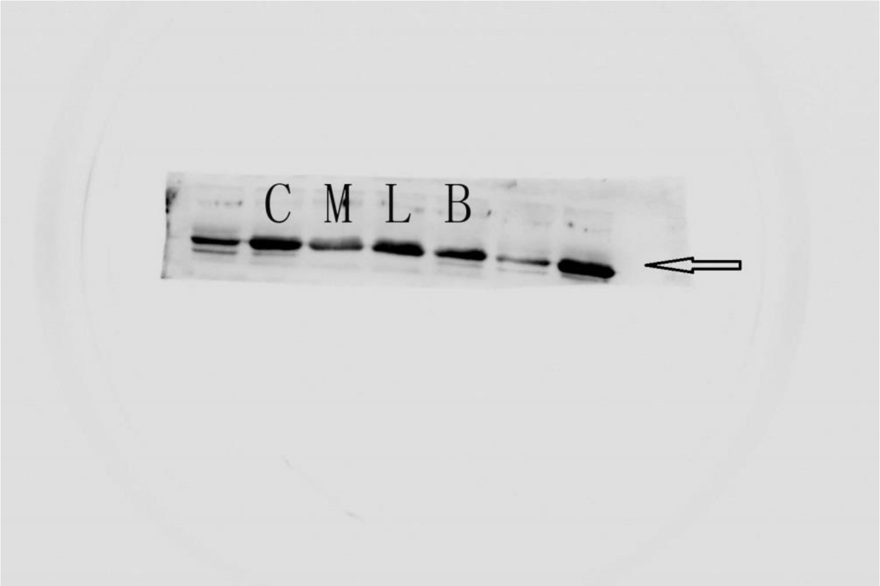

18. The full-length gels: Fig.11-LXR-α （β-actin）

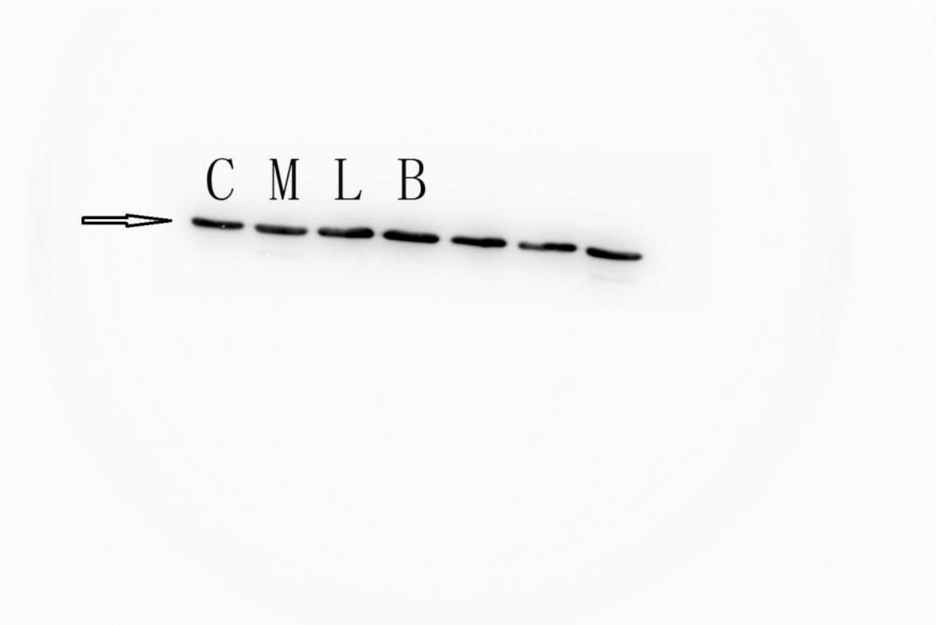

Supplement: Supplementary Information [file srep40523-s1.pdf]
